# Cardiomyopathies and myocardial disorders in Africa: present status and the way forward

**Published:** 2013-10

**Authors:** Ayodele Falase, Okechukwu Ogah

**Affiliations:** Division of Cardiology, Department of Medicine, University College Hospital, Ibadan, Nigeria

## Dear Sir

We are grateful to Prof Bongani Mayosi^1^ for his comments on our article ‘Cardiomyopathies and myocardial disorders in Africa’.^2^ We thank him for bringing to our attention previous publications from Africa on left ventricular non-compaction and ion channelopathies. These publications were not available to us when we wrote the article. It however shows that these diseases also exist in Africa.

Interestingly we have, since our publication, encountered a case of Brugada syndrome in our practice, although we are yet to report the case. We therefore agree that many of the myocardial diseases reported from the rest of the world probably also exist here in Africa.

The current problem however is how to define and classify the cardiomyopathies. One of us (AF) was part of the group that first proposed the name cardiomyopathy for this group of diseases.^3^,^4^ We also classified them at the time, based on existing knowledge. The key principle we agreed upon at our meeting was to name any disease of the myocardium according to the disease that caused it. Those that we did not know the cause or causes of were the ones we regarded as cardiomyopathies.

In our view, subsequent attempts at classifying these disorders of the myocardium have disregarded this basic principle and have labelled virtually all diseases of the myocardium as cardiomyopathy. Such assumptions have made classification of the diseases more complex and difficult to use in routine clinical practice. They also ignore geographic differences in the causation and presentation of the diseases, especially in Africa where the problem of the cardiomyopathies is most profound.

We do not think that the current European Society of Cardiology classification is suitable for clinicians working in Africa.^5^ We therefore believe that it is time for Africa to develop its own classification based on the realities on the continent.

Our classification was designed to trigger a debate in Africa towards this end and in so doing we have continued to maintain that basic philosophy of calling every disorder of the myocardium by the disease that caused the disorder. We have through that avoided the unending controversies surrounding the definition of cardiomyopathy and have attempted to bring all the disorders of the myocardium under one classification. We agree that the Pan-African Society of Cardiology (PASCAR) should take further steps to either adopt or modify this classification to suit the clinical realities of Africa.
